# L’acromégalie du sujet âgé: quelles particularités?

**DOI:** 10.11604/pamj.2017.27.169.11518

**Published:** 2017-07-04

**Authors:** Nadia Anoun, Hanan El Ouahabi

**Affiliations:** 1 Service d’Endocrinologie, Diabétologie et Nutrition, CHU Hassan II de Fès, Maroc; 2 Equipe Sciences des Médicaments, Centre Médical de Recherche Biomédicale et Translationnelle, Faculté de Médecine et de Pharmacie de Fès, Maroc

**Keywords:** Acromégalie, sujet âgé, chirurgie hypophysaire, pronostic, Acromegaly, aging population, pituitary surgery, prognosis

## Abstract

Les adénomes somatotropes de la personne âgée sont rares, et caractérisés par un retard diagnostique, un tableau clinique peu bruyant. Leurs critères diagnostiques rejoignent ceux des patients plus jeunes. La chirurgie, si possible, reste le traitement de choix de l’acromégalie du sujet âgé. Les analogues de la somatostatine ont montré leur efficacité dans le traitement de ces patients. Le pronostic des patients acromégales est inversement corrélé à l’âge du patient, à la durée de la maladie, au dernier taux de GH sous traitement. L’âge est un déterminant majeur de la mortalité en dehors de l’évolutivité de l’acromégalie. Nous rapportons trois observations de patientes acromégales âgées respectivement de 75, 70 et 66 ans avec une revue de la littérature.

## Abstract (en)

Somatotroph adenomas are rare in the aging population. Diagnosis of somatotroph adenomas is often long delayed and they are characterized by atypical clinical picture. Their diagnostic criteria are similar to those used for younger patients. Surgery, if possible, is the treatment of choice for acromegaly in the elderly. Somatostatin analogues have shown to be effective in these patients. Prognosis is inversely correlated with patient’s age, duration of disease and last GH level under treatment. Beside evolution of disease, age is a major determinant of mortality. We report three cases of elderly patients with acromegaly aged 75, 70 and 66 years respectively with a literature review.

## Introduction

Les adénomes hypophysaires des personnes âgées de plus de 65 ans représentent moins de 10% de tous les adénomes hypophysaires, dont 80% sont non fonctionnels. Les adénomes somatotropes restent très rares. Leur présentation clinique est dominée par le déficit visuel, suivie des symptômes endocriniens. Leurs critères diagnostiques et leur prise charge rejoignent ceux des patients plus jeunes. Nous rapportons trois observations de patientes acromégales âgées respectivement de 75, 70 et 66 ans avec une revue de la littérature.

## Patient et observation

**Figure 1:**
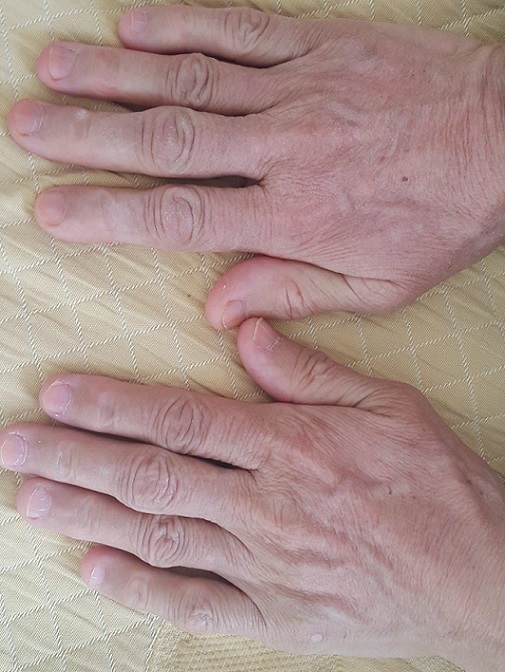
Aspect des mains (1A) et des pieds (1B) chez une patiente acromégale

**Figure 2:**
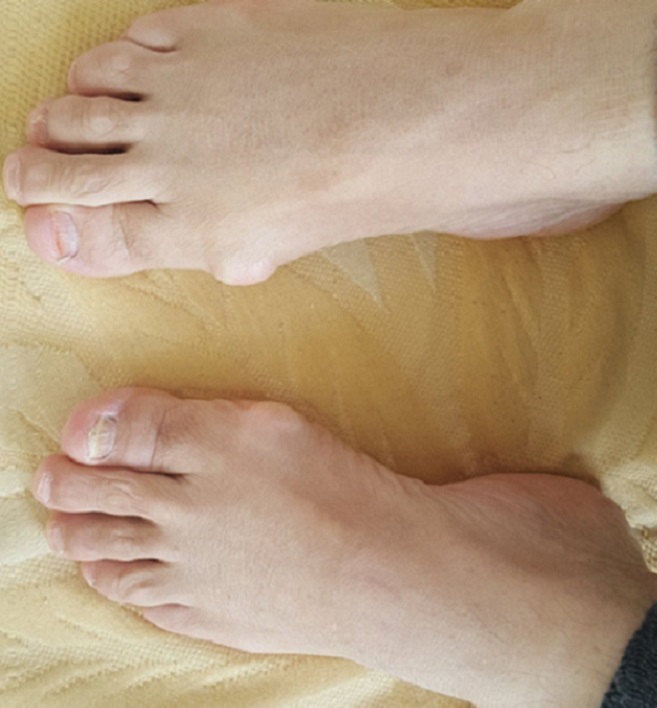
Coupe sagittale d’une IRM hypothalamo-hypophysaire montrant une anomalie de signal anté-hypophysaire médiane, sans signes d’envahissement, mesurant 12 x 10 x 7,6 mm dans ses plus grands axes

Une patiente âgée de 75 ans consulte pour un goitre nodulaire, on trouve à l’examen clinique un syndrome dysmorphique acro-facial évoquant une acromégalie (Figure 1), l’interrogatoire révèle des céphalées frontales avec vertige et brouillard visuel depuis 1 an. Le diagnostic suspecté fut confirmé par un taux d’IGF élevé à 2,5 fois la normale, avec à l’IRM hypothalamo-hypophysaire un macroadénome hypophysaire de 12mm (Figure 2). Le bilan de retentissement a mis en évidence une hypothyroïdie centrale, une altération bilatérale du champs visuel plus marquée en nasal inférieur, au bilan d´organomégalie un goitre nodulaire, et sur le plan métabolique une intolérance aux hydrates de carbone. La patiente fut perdue de vue par la suite.

Une patiente âgée de 70 ans, consulte pour des céphalées et des polyarthralgies, l’examen clinique trouve un faciès acromégaloïde. Le diagnostic fut confirmé devant des IGF1 à 3,3 fois la normale avec un macroadénome hypophysaire de 11mm, sans retentissement endocrinien mais avec une altération du champs visuel à droite, un bilan d´organomégalie normal, sans complications métaboliques. La patiente a bénéficié d’une ablation de son adénome à GH par voie transphénoidale avec des suites post-opératoires simples. L’évaluation post-opératoire à 3 mois était en faveur de la guérison (normalisation de l’IGF1 et absence de résidu sur l’imagerie hypophysaire).

**Figure 3:**
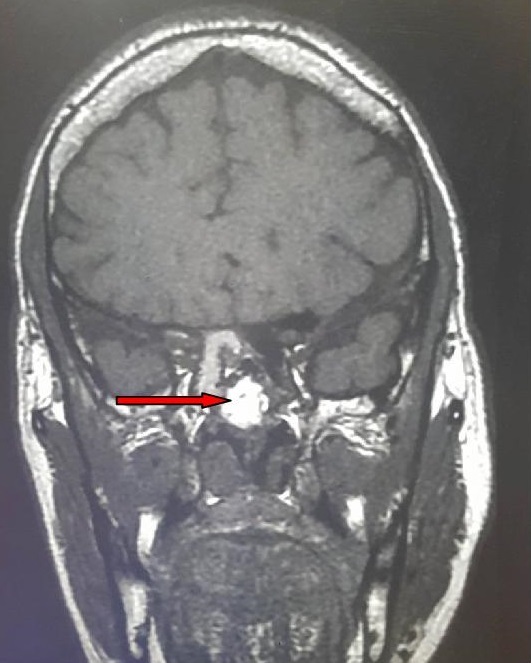
Coupe coronale d’une IRM hypothalamo-hypophysaire montrant un macroadénome hypophysaire mesurant 15mm de hauteur latéralisé droite

Une patiente âgée de 66 ans consulte pour un syndrome dysmorphique acro-facial classique d’une acromégalie, avec un taux d’IGF1 à 2,78 fois la normale et à l’IRM hypothalamo-hypophysaire un macroadénome hypophysaire de 15mm (Figure 3), avec comme retentissement endocrinien une insuffisance corticotrope et thyréotrope et endocrânien une hémianopsie temporale droite, au bilan d´organomégalie un goitre nodulaire et sur le plan métabolique un diabète et une HTA secondaires. La patiente a été traitée chirurgicalement par voie transphénoidale avec des suites post-opératoires simples. Une guérison fut déclarée après l’évaluation à 3 mois du post-opératoire montrant une normalisation de l’IGF1 et l’absence de résidu sur l’IRM hypothalamo-hypophysaire.

## Discussion

L´acromégalie est un trouble neuroendocrinien insidieux résultant généralement d’une hypersécrétion de GH par un adénome hypophysaire. Elle se voit principalement au cours de la 3^ème^ - 4^ème^ décennie de vie, mais les études récentes ont montré une incidence assez élevée d´adénomes hypophysaires chez les personnes âgées [1], probablement en raison de la proportion croissante de ces derniers.

La présentation clinique d’un adénome hypophysaire chez la personne âgée peut être fort atypique. Les changements endocriniens physiologiques dus à l’âge, les fréquentes comorbidités, une altération du champs visuel par dégénérescence sénile peuvent brouiller ou retarder le diagnostic, ce qui a été appuyé par les séries publiées dans la littérature [2, 3]. D’autres explications sont aussi évoquées, parmi lesquelles une agressivité tumorale moins importante à cet âge en lien avec des taux de GH et d’IGF1 moins élevés que chez l’adulte jeune [4], en effet les sujets âgés sont plus susceptibles d´avoir des microadénomes que les jeunes patients avec cette hypothèse . En outre, les variations des facteurs endocrines et paracrines liées à l’âge, tels que les hormones sexuelles, les facteurs métaboliques ou la vascularisation de la tumeur pourraient être responsables de cette différence [5]. Il a été également rapporté dans la littérature que l´insuffisance pituitaire est plus probable chez les jeunes patients [5]. Une relation entre la taille de la tumeur et le degré de l insuffisance hypophysaire a été suggérée [6].

Sur le plan thérapeutique, la chirurgie est recommandée en première intention et ne pose habituellement que peu de risques de morbidité [7, 8]. L’âge avancé, l’accumulation de facteurs de risque et de tares viscérales augmentent cependant la vulnérabilité de ces sujets. Les données de mortalité impliquent pourtant de traiter avec les mêmes objectifs les patients les plus fragiles. Il convient de signaler que la mise à disposition des analogues de la somatostatine depuis les années 1990 a considérablement enrichi les outils de la prise en charge de l’acromégalie. En effet, ce traitement permet parfois de s’affranchir de la chirurgie pour contrôler la tumeur et sa sécrétion hormonale excessive [9, 10]. Ce fait plaide donc pour un recours moins systématique à la solution chirurgicale lorsque celle-ci paraît plus délicate ou dangereuse.

L’utilisation de la radiothérapie devient actuellement plus rare. La disponibilité des alternatives médicales et la responsabilité potentielle de la radiothérapie dans la majoration des maladies cérébro-vasculaires [11] expliquent en partie la prudence vis-à-vis de ce moyen thérapeutique. Si des indications de la radiothérapie persistent [12], sa prescription est néanmoins en recul [13]. Il est à noter que les patients âgés semblent également avoir un taux de guérison plus élevé après la chirurgie et en réponse au traitement par analogue de la somatostatine [5].

## Conclusion

Comme l’espérance de vie augmente dans notre pays, une augmentation de la prévalence de l’acromégalie chez les sujets âgés devrait être attendue. La réduction de l’espérance de vie chez les acromégales non traités, soulignée fortement dans la littérature [4, 6], incite à une plus grande rigueur dans les objectifs thérapeutiques et le contrôle des facteurs de risque. L’arrivée sur le marché de traitements modernes et très actifs justifie l’évaluation de nos pratiques afin d’assurer à cette population particulière une prise en charge optimale.

## Conflits d’intérêts

Les auteurs ne déclarent aucun conflit d’intérêts.
